# Identification and Characterization of the Regulatory Particle of Proteasome 19S and Its Correlation with Proteasome 26S in Trophozoites of *Naegleria fowleri*

**DOI:** 10.3390/microorganisms14061277

**Published:** 2026-06-05

**Authors:** Itzel Citlalli Rubio-Gutiérrez, Angélica Silva-Olivares, Paula Guzmán-Téllez, Rosa María del Ángel, Abigail Betanzos-Fernández, Sirenia González-Pozos, Jesús Serrano-Luna

**Affiliations:** 1 Department of Cell Biology, Center for Research and Advanced Studies of the National Polytechnic Institute, Av. IPN 2508, Mexico City 07360, Mexico; itzel.rubio@cinvestav.mx; 2 Department of Infectomics and Molecular Pathogenesis, Center for Research and Advanced Studies of the National Polytechnic Institute, Av. IPN 2508, Mexico City 07360, Mexico; 3 Laboratory of Immunoregulation, Department of Immunology, National School of Biological Sciences of the National Polytechnic Institute, Av. Luis Enrique Erro S/N, Mexico City 07738, Mexico; 4 National Laboratories for Experimental Services (LANSE), Center for Research and Advanced Studies of the National Polytechnic Institute, Av. IPN 2508, Mexico City 07360, Mexico

**Keywords:** *Naegleria*, regulatory particle, 19S proteasome, 26S proteasome, purification

## Abstract

The genus *Naegleria* comprises free-living amoebae characterized as amphizoic and ubiquitous microorganisms. *Naegleria fowleri* is the only species pathogenic to humans, causing primary amebic meningoencephalitis. The 26S proteasome represents the principal catalytic complex responsible for the degradation and recycling of intracellular proteins in eukaryotic cells. This complex consists of the 20S and 19S proteasome subunits, with the latter involved in the recognition and processing of ubiquitinated proteins and their delivery to the degradation site. Although the 26S proteasome has been characterized in various pathogenic protozoa, only the 20S proteasome has been studied within the genus *Naegleria*. The objective of this study was to demonstrate the presence of 19S subunits in *N. fowleri*. Bioinformatics analyses were employed to evaluate the presence and homology of non-ATPase subunits (Rpn10, Rpn11, and Rpn13) and ATPase subunits (Rpt2, Rpt3, and Rpt5). Additionally, the presence, localization, and correlation of 19S proteasome proteins with the 20S proteasome were assessed using experimental approaches. The results indicate that *N. fowleri* possesses proteins corresponding to the 19S proteasome, which, together with the 20S core particle, contribute to the formation of the 26S proteasome.

## 1. Introduction

Free-living amoebae (FLA) comprise a group of protozoa that are both ubiquitous in the environment and capable of adopting an amphizoic lifestyle. Several genera within this group—such as *Acanthamoeba* spp., *Balamuthia mandrillaris*, *Sappinia pedata*, and *Naegleria* spp.—are recognized as pathogenic or opportunistic microorganisms capable of causing infections in humans and other animals [1,2,3]. The genus *Naegleria* is predominantly found in natural freshwater environments (rivers, lakes, and streams) or even in various human-made settings such as swimming pools and untreated domestic water sources [4]. Within this genus, *N. fowleri* is the only species pathogenic to humans, responsible for causing a central nervous system (CNS) infection known as primary amebic meningoencephalitis (PAM). This disease is highly fatal with an estimated mortality rate of approximately 98%. To date, no specific treatment exists, and the only drug demonstrating limited efficacy—effective in roughly 2% of cases—is amphotericin B (AmB) [2,5,6,7,8].

In eukaryotic organisms, the ubiquitin–proteasome system plays a crucial role in the degradation of misfolded, damaged, or short-lived regulatory proteins [9,10]. The 26S proteasome is a multicatalytic complex regarded as the central protease in the intracellular protein degradation and recycling pathway. Its activity is essential for key cellular processes (proliferation, differentiation, cell cycle regulation, and apoptosis). This complex is composed of two distinct subcomplexes: the core protease, known as the 20S proteasome, and the regulatory particle (RP), referred to as the 19S proteasome [11,12,13,14]. The 19S proteasome consists of at least 19 subunits organized into two main subcomplexes: the base and the lid. The base includes six ATPase subunits of the AAA family (ATPases associated with a variety of cellular activities), designated Rpt1–Rpt6, along with three non-ATPase subunits, Rpn1, Rpn2, and Rpn10. The lid comprises the remaining non-ATPase subunits, Rpn3, Rpn5–Rpn9, and Rpn11–Rpn12 [11,15]. The 19S proteasome is responsible for several key functions, including the recognition and unfolding of ubiquitinated proteins, the removal of covalently attached ubiquitin, the opening of the α-ring, and the targeting of substrate peptides toward the core protease for cleavage [11,12].

It has been reported that the genomes of pathogenic microorganisms encode proteasomes that are structurally and functionally analogous to those present in mammals [16]. In parasitic protozoan infections, inhibition of proteasome proteolytic activity has been shown to prevent morphological changes and cellular proliferation, both of which are critical during the infectious stage. Consequently, the study, purification, and characterization of the proteasome in various genera of parasitic protozoa, including *Leishmania* spp., *Trypanosoma* spp., *Entamoeba* spp., and *Plasmodium* spp., are of significant interest, as the proteasome represents a potential therapeutic target [10,17]. In this study, bioinformatics analyses were employed to investigate the presence and structural characteristics of homologous protein sequences corresponding to 19S proteasome subunits. Additionally, several subunits were detected using dot blot assays, and their cytoplasmic localization was determined through immunofluorescence and confocal microscopy. The presence of the 26S proteasome in *N. fowleri* trophozoites was further confirmed using mass spectrometry, confocal microscopy, and transmission electron microscopy.

## 2. Materials and Methods

### 2.1. In Silico Analysis of the 19S Proteasome Subunits in Naegleria fowleri

The bioinformatics analysis of the putative 19S regulatory particle subunits in *N. fowleri* was carried out using as references the amino acid sequences previously reported for *Homo sapiens* in the KEGG Orthology (KO) database (https://www.kegg.jp/pathway/map03050; accessed on 19 November 2020) and the proteasome map (ref: 03050). The analyzed subunits included the AAA ATPase components Rpt2, Rpt3, and Rpt5 (ref: K03062, K03063, and K03065), as well as the non-ATPase subunits Rpn10, Rpn11, and Rpn13 (ref: K03029, K03030, and K06691). To obtain the corresponding sequences from *N. fowleri*, amino acid alignments were performed using the BLAST-P tool with default parameters on the AmoebaDB server (https://amoebadb.org/amoeba/app; accessed on 23 November 2020). Based on the recovered sequences, homology levels between *N. fowleri* and *H. sapiens* subunits were determined. Comparative analyses were performed with the NCBI BLAST-P program (https://blast.ncbi.nlm.nih.gov/Blast.cgi?PAGE=Proteins; accessed on 30 November 2020) using predefined parameters, applying the significance of e-values and the percentages of identity and similarity between *N. fowleri* sequences and those of the reference species as criteria.

Three-dimensional structural models of the *N. fowleri* subunits were generated using the Structure Prediction tool of the RaptorX server (http://raptorx.uchicago.edu/ContactMap/ accessed on 8 December 2020). Additionally, three-dimensional alignments and comparative analyses between *H. sapiens* and *N. fowleri* subunits were conducted using the homology percentage provided by the MatchMaker tool of UCSF Chimera 1.11.3 and the similarity values obtained from the DALI program (http://ekhidna2.biocenter.helsinki.fi/dali/ accessed on 14 January 2021).

### 2.2. Amoeba and Cell Cultures

*N. fowleri* (ATCC 30808) was maintained in axenic culture using 2% (*w*/*v*) Bactocasitone medium (Becton Dickinson, Franklin Lakes, NJ, USA) supplemented with 10% (*v*/*v*) fetal bovine serum (Biowest, Nuaillé, France). Trophozoites were harvested at 48 h during the logarithmic growth phase at 37 °C. The human hepatocyte-derived carcinoma cell line HUH-7 (kindly provided by Dr. Del Ángel, CINVESTAV-IPN, Mexico City, Mexico) was cultured to confluence in Advanced DMEM (Gibco, Thermo Fisher Scientific, Waltham, MA, USA) supplemented with 200 mM glutamine, penicillin (5 × 10^4^ U/mL), streptomycin (50 μg/mL) (Invitrogen, Carlsbad, CA, USA), 7% fetal bovine serum, and 1 mL/L amphotericin B (Fungizone, Gibco, Thermo Fisher Scientific, Waltham, MA, USA) under a humidified atmosphere containing 5% CO_2_ at 37 °C.

### 2.3. Preparation of N. fowleri and HUH-7 Cell Extracts

Axenically cultured *N. fowleri* trophozoites were incubated on ice for 15 min to facilitate detachment from the culture flask. Trophozoites were then collected and washed with PBS (pH 7.2) by centrifugation at 375× *g* for 15 min twice. The resulting pellet was resuspended in an equal volume of lysis buffer (150 mM NaCl, 2% Triton X-100 [*v*/*v*], 50 mM Trizma base, pH 8.4; Sigma-Aldrich, St. Louis, MO, USA) and a protease inhibitor cocktail (cOmplete™, MERCK, Darmstadt, Germany). Samples were subjected to seven cycles of freeze–thawing to ensure complete lysis. Total protein extracts from HUH-7 cells were prepared from monolayers at ~90% confluence. Cells were washed once with PBS (pH 7.2) and harvested by scraping in radioimmunoprecipitation assay (RIPA) buffer (25 mM Tris-HCl, pH 7.6; 150 mM NaCl; 5 mM EDTA; 1% Triton X-100; 1% sodium deoxycholate; 0.1% SDS) supplemented with the cOmplete™ protease inhibitor cocktail. Extracts were incubated on ice for 5 min and subsequently centrifuged at 12,000× *g* for 10 min. The supernatant containing soluble proteins was collected and stored for subsequent analyses.

### 2.4. Detection of Proteasome 19S Subunits by Western Blot and Dot Blot Assay

Total protein extracts from *N. fowleri* and HUH-7 cells were quantified using the Bradford assay [18] and adjusted to 40 μg of protein per HUH-7 cell sample and 60 μg of protein per *N. fowleri* sample; they were then resolved by 12% SDS-PAGE and transferred to a 0.45 µm nitrocellulose membrane (Bio-Rad, Berkeley, CA, USA) for 120 min at 400 mA and 4 °C. After transfer, the membranes were blocked with 5% skim milk/TBS for 2 h at room temperature with constant agitation, followed by two washes of 10 min each with 0.05% (*v*/*v*) TBS-T. Membranes were then incubated overnight at 4 °C with continuous shaking using the following primary antibodies: mouse monoclonal anti-Proteasome 19S S5A/ASF (1:4000, ABCAM, Cambridge, UK) and mouse monoclonal anti-PSMC6 (1:4000, ABCAM, Cambridge, UK). Following incubation, membranes were washed twice for 10 min with 0.05% TBS-T and incubated for 2 h at room temperature with goat anti-mouse IgG H&L (HRP) 1:4000 (ABCAM, Cambridge, UK). Membranes were then washed five times for 10 min each with 0.05% TBS-T and developed using a luminol-based chemiluminescent reagent (Santa Cruz Biotechnology, Dallas, TX, USA). Protein signals were visualized and analyzed using the Odyssey FC imaging system (LI-COR) and Image Studio software version 5.2.

For dot blot assays, 25 μg of protein per sample was spotted onto 0.45 μm nitrocellulose membranes (Bio-Rad, Berkeley, CA, USA). Membranes were blocked with 5% (*w*/*v*) skim milk for 2 h at room temperature with constant agitation, followed by two washes of 10 min each with 0.05% (*v*/*v*) PBS-T. Membranes were then incubated overnight at 4 °C with continuous shaking using the rabbit recombinant monoclonal anti-PSMD14 (1:4000, ABCAM, Cambridge, UK) or mouse anti-actin monoclonal antibody (1:500, Santa Cruz Biotechnology, Dallas, TX, USA). Following incubation, membranes were washed twice for 10 min with 0.05% PBS-T and incubated for 2 h at room temperature with the appropriate secondary antibody: goat anti-mouse IgG H&L (HRP) or goat anti-rabbit IgG H&L (HRP), both at 1:4000 (ABCAM, Cambridge, UK). The membranes were subsequently washed five times for 10 min each with 0.05% PBS-T. Signal detection was then performed following the same procedure described for the Western blot analysis.

### 2.5. Immunolocalization of ATPase and Non-ATPase Subunits of the 19S Proteasome in N. fowleri

Coverslips were placed in 24-well plates and seeded with approximately 200,000 *N. fowleri* trophozoites or HUH-7 cells, which were incubated for 24 h at 37 °C, with HUH-7 cells maintained in a 5% CO_2_ atmosphere. Cells and amoebae were fixed with 4% (*w*/*v*) paraformaldehyde (Sigma-Aldrich, St. Louis, MO, USA) for 15 min, washed twice with sterile PBS, and permeabilized for 10 min with 0.2% (*v*/*v*) Triton X-100. Samples were then blocked for 1 h at 37 °C with 1% (*w*/*v*) albumin (Sigma-Aldrich, Darmstadt, Germany) in PBS and washed twice with PBS. Coverslips were incubated for 2 h at 37 °C with primary antibodies: mouse monoclonal anti-Proteasome 19S S5A/ASF (1:100, ABCAM, Cambridge, UK), rabbit recombinant monoclonal anti-PSMD14 (1:100, ABCAM, Cambridge, UK) or mouse monoclonal anti-PSMC6 (1:100, ABCAM, Cambridge, UK). Samples were then labeled with the secondary antibody, goat anti-mouse IgG H&L (Alexa Fluor^®^ 488, 1:200, ABCAM, Cambridge, UK) and FITC-goat anti-rabbit IgG (H + L) (1:200, Invitrogen, Carlsbad, CA, USA) for 1 h at 37 °C. Nuclei were stained with 4′,6-diamidino-2-phenylindole (DAPI, 1 μg/mL, Thermo Fisher Scientific, Waltham, MA, USA) for 10 min at room temperature. Coverslips were mounted using Vectashield (Vector Laboratories, Newark, CA, USA), and images were acquired with a confocal microscope (Carl Zeiss LSM-900; Zeiss, Jena, Germany).

To evaluate the colocalization of 19S and 20S proteasome subunits, the same procedure was followed with the addition of the anti-proteasome 20S C2/HC2 antibody (1:100, ABCAM, Cambridge, UK) and anti-20S proteasome β5-subunit (1:100, Sigma-Aldrich, Darmstadt, Germany) during primary incubation, followed by labeling with goat polyclonal to mouse IgG H&L (Alexa Fluor^®^ 647, 1:200, ABCAM, Cambridge, UK) and donkey anti-rabbit IgG H&L (Alexa Fluor^®^ 647, 1:200, ABCAM, Cambridge, UK) as the secondary antibody.

### 2.6. Purification of the 26S Proteasome

Amoebae were grown in four 75 cm^2^ flasks for 48 h at 37 °C (*N. fowleri*). HUH-7 cells were also grown in four flasks to a confluence of 95–100%. Subsequently, the amoebae and cells were detached from the flasks and centrifuged at 7227× *g* for 15 min. The resulting pellet was resuspended in 10 mL of 50 mM Tris-HCl (pH 7.4). A second wash was performed under the same centrifugation conditions, and the pellets were then resuspended in 5 mL of standard buffer (10% glycerol, 50 mM Tris-HCl, pH 7.4, 10 mM MgCl_2_, 0.25 M sucrose, 4 mM ATP, and 1 mM DTT). The pellet was subjected to sonication three times at 100 W using 10 s pulses, repeated four times. Thereafter, the sample was centrifuged at 7227× *g* for 15 min to remove cell debris. Samples were subsequently ultracentrifuged at 71,000× *g* (Optima MAX-XP ultracentrifuge, Beckman Coulter, Pasadena, CA, USA) for one hour, and the supernatant was centrifuged again at 105,000× *g* for five hours. The final pellet was resuspended in 100 µL of standard buffer. This final solution was used in the subsequent experimental techniques (proteomics and electron microscopy). To maintain the integrity of the complete 26S proteasome, stabilization was required throughout the purification process. Therefore, a standard buffer supplemented with ATP was added at each corresponding step of the purification technique. Subsequently, to ensure appropriate control of the purification procedure, SDS–PAGE analysis was performed (Supplementary Figure S1) to confirm the presence of proteins.

### 2.7. In-Solution Sample Preparation for Mass Spectrometry Analysis

Mass spectrometry analysis was performed in the Genomics, Proteomics and Metabolomics Unit at Cinvestav. First, 50 µg of protein with the standard buffer for each sample was precipitated using methanol/chloroform in a ratio of 4:1. The resulting pellets were resuspended using 200 µL of lysis buffer (1 mM EDTA, 20 mM HEPES, 8 M urea). Then, samples were sonicated using Birruptor Pico equipment (Diagenode, Liège, Belgium) for 5 cycles of 20 s ON and 30 s OFF. Afterward, samples were reduced with 10 mM DTT in 100 mM ammonium bicarbonate (ABC) for 30 min at 56 °C and alkylated with 15 mM iodoacetamide (IAA) in 100 mM ABC at room temperature covered with light, and then diluted with 20 mM HEPES (pH 8) to reach a concentration of 2 M urea. Subsequently, protein in each sample was enzymatically digested with proteomic-grade trypsin (Promega, Madison, WI, USA) at 37 °C overnight in a ratio of 1:50 (enzyme: protein). Finally, samples were desalted with PIERCE^®^ Spin Columns C18 (Thermo Fisher Scientific, Waltham, MA, USA) and eluted with 20 µL of 0.1% formic acid (FA) in 50% acetonitrile (ACN) and with 0.5% FA in 70% ACN twice. Finally, samples were kept at −20 °C until LC-MS analysis.

### 2.8. Mass Spectrometry-Based Proteomics

Chromatographic and spectrometric methods were applied according to the analysis by [19]. Mass spectrometry (MS) analysis was performed on a Synapt G2-Si mass spectrometer (Waters Corp, Milford, MA, USA). Tryptic peptides were separated on a HSS T3 C18 Column (Waters Corp, Milford, MA, USA); 75 μm × 150 mm, 100 Å pore size, 1.8 μm particle size; by a UPLC ACQUITY M-Class (Waters Corporation, Milford, MA, USA) using mobile phase A (0.1% FA in H_2_O) and mobile phase B (0.1% of FA) in ACN under the following gradient: 7% B (0 min), 40% B (121.49 min), 85% B (123.15–126.46 min), and 7% B (129–130 min), at a flow rate of 400 nL min^−1^ and 45 °C. The spectral data were acquired using nano-electrospray ionization and ion mobility separation (IM-MS) using the full-scan DIA approach through the high-definition multiplexed MS/MS (HDMSE) mode [20,21]. Parameters for the ionization source were set to the following values: 2.75 kV in the sampling capillary, 30 V in the sampling cone, 30 V in the source offset, 70 °C for the source temperature, 0.5 bar for the nanoflow gas, and 150 L h^−1^ for the purge gas flow. Two chromatograms were acquired (low- and high-energy chromatograms) in positive mode in a range of *m*/*z* 50–2000 with a scan time of 500 ms. For the high-energy chromatograms, the precursor ions were fragmented in the transfer cell using a collision-induced dissociation (CID) energy ramp from 19 to 55 eV. Calibration of the Synapt G2-Si was performed with [Glu1]-Fibrinopeptide fragments via the precursor ion [M + 2H]2+ = 785.84261, with a fragmentation of 32 eV and an error of less than 1 ppm in all MS/MS measurements.

### 2.9. Database Search

The *.raw files containing MS and MS/MS spectra were analyzed and quantified with the software Progenesis QI for Proteomics v4.2 (Nonlinear Dynamics, Newcastle, UK) [22] using a target decoy strategy to detect false positives [23,24] against *H. sapiens* (downloaded from UniProt on 27 February 2024, UP000005640, 104,557 protein sequences) and *N. fowleri* (downloaded from UniProt on 15 May 2024, UP000444721, 13,596 protein sequences) *.fasta databases. Parameters used during the database search were set as follows: trypsin as a cut enzyme and one missed cleavage allowed; carbamidomethyl © as a fixed modification and amidation (N-term), deamidation (N, Q), oxidation (M), and phosphoryl (S, T, Y) as variable modifications; automatic peptide and fragment tolerance, minimum fragment ion matches per peptide: 2, minimum fragment ion matches per protein: 5, minimum peptide matches per protein: 1, and a false discovery rate (FDR) ≤ 1%. All false positive identifications (reversed proteins) were eliminated from the study, and the quantitative results at the protein and peptide levels were exported as *.csv files for subsequent analysis.

### 2.10. Transmission Electron Microscopy (TEM)

A final volume of 100 μL from each sample pellet was used to analyze the structure of the 26S proteasome by transmission electron microscopy. Samples were diluted 1:5000 and applied to copper grids for approximately 1 min, after which excess liquid was removed. Grids were then stained twice with 2% uranyl acetate for approximately 1 min each. The negatively stained samples were examined using a JEOL JEM-1400 electron microscope (JEOL Ltd., Tokyo, Japan) at a magnification of 50,000×.

## 3. Results

### 3.1. Naegleria fowleri Has Six Putative ATPase and Non-ATPase Subunits of the 19S Proteasome

**Figure 1:**
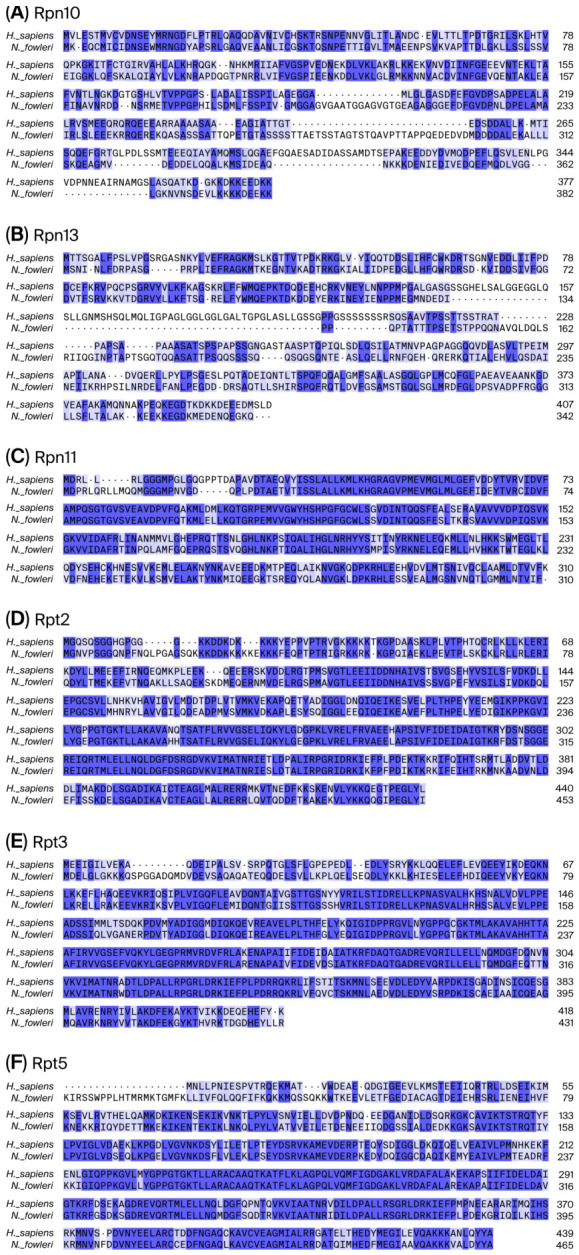
Multiple alignments of ATPase and non-ATPase subunits of 19S proteasome in *N. fowleri***.** (**A**,**B**) Among the proteins analyzed for homology, the proteasome receptors show a lower degree of conservation, exhibiting a greater number of gaps. (**C**) Rpn11 is a highly conserved protein essential for the proper functioning of the proteasome. (**D**–**F**) The ATPase subunits are highly conserved according to the homology obtained between the amoeba and *H. sapiens*.

The 19S regulatory particle is responsible for regulating the access of substrates to the 20S core particle, or proteasome. It is composed of AAA ATPase and non-ATPase subunits. The in silico analysis allowed the identification and characterization of six putative subunits of the 19S regulatory particle in *N. fowleri* (Figure 1). The results indicate the presence of three AAA ATPase subunits—Rpt2, Rpt3, and Rpt5—associated with the mechanism responsible for opening the gate of the 20S proteasome. Additionally, three non-ATPase subunits were identified: Rpn10 and Rpn13, both implicated in the recognition of polyubiquitinated substrates and functioning as proteasome receptors, and Rpn11, which appears to act as a deubiquitinating enzyme responsible for removing the covalent bond between ubiquitin and the target protein. When aligning and analyzing the sequences with the Clustal W algorithm (Jalview), the three non-ATPase subunits (Figure 1A–C) of the 19S proteasome in the amoeba showed highly conserved regions (dark blue) with respect to the sequences found in *H. sapiens*. Among them, the Rpn11 subunit (Figure 1C) exhibited the greatest sequence conservation, whereas Rpn10 and Rpn13 (Figure 1A,B) displayed a smaller number of conserved amino acids. In contrast, the three ATPase subunits Rpt2, Rpt3, and Rpt5 (Figure 1D–F) showed a highly conserved sequence relative to *H. sapiens*. Regions with the lowest degree of conservation across all sequences were observed in light blue. Specifically, a dark blue coloration indicates that the amino acid matches the *H. sapiens* amino acid at that position in the sequence, indicating high conservation or identity. In addition, light blue denotes a residue that does not match the corresponding residue in *H. sapiens* but shares a positive score in the BLOSUM62 substitution matrix, suggesting that the substitution is conserved. This is because the amino acids have similar physicochemical properties, and the protein function is probably not compromised by the substitution. Furthermore, the dashes indicate gaps that have been inserted to maximize the similarity between the sequences. These represent evolutionary insertion or deletion events that have occurred in one of the sequences since the proteins (or organisms) diverged from a common ancestor. Overall, these results indicate that the ATPase subunits present a higher percentage of peptide sequence conservation between the model organism and *N. fowleri* compared with the non-ATPase subunits.

Based on the recovered sequences for the ATPase (Rpt2, Rpt3, and Rpt5) and non-ATPase (Rpn10, Rpn11, and Rpn13) subunits, the percentages of similarity and identity between *N. fowleri* and *H. sapiens* were calculated using BLAST-P analysis. According to the results, the ATPase subunits and Rpn11 showed identities and similarities greater than 70%. Notably, the e-values obtained for the Rpt subunits were 0.0. In the case of ubiquitin receptors, the percentages were lower; however, similarity and identity values were still higher than 50%, with the exception of the identity percentage for Rpn10 (Table 1). Together with the previous findings, these results demonstrate that *N. fowleri* possesses putative proteins corresponding to the six analyzed subunits and that these sequences contain widely conserved regions. Nevertheless, the most conserved subunits are the ATPase components and the deubiquitinating enzyme Rpn11.

### 3.2. The Putative Non-ATPase and ATPase Subunits of the 19S Proteasome of N. fowleri Present Three-Dimensional Arrangements

**Figure 2:**
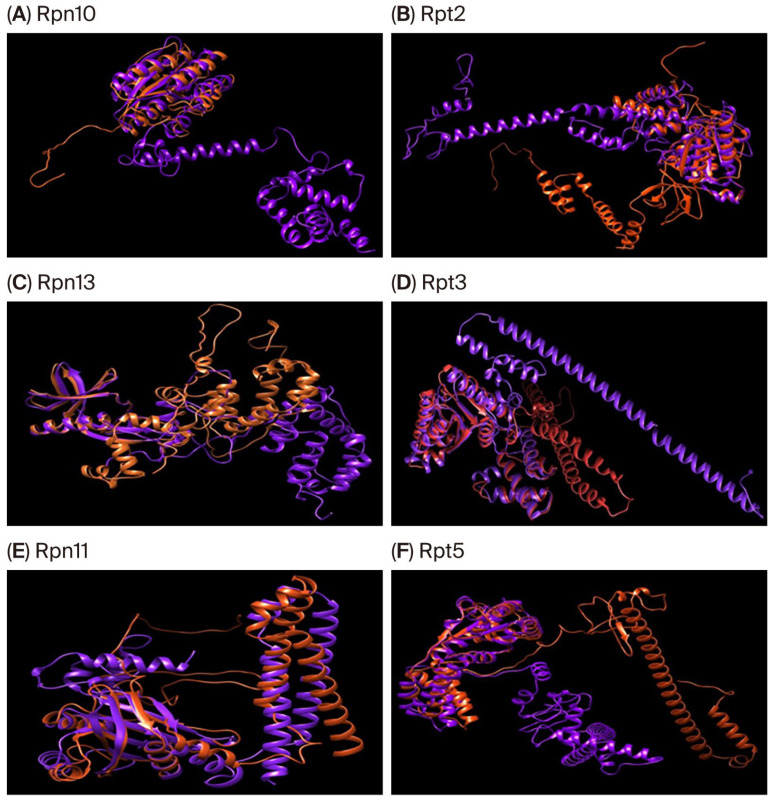
Predicted structures of ATPase and non-ATPase subunits. The three-dimensional models indicate the proteins of *N. fowleri* (purple) and *H. sapiens* (orange). (**A**,**B**) Structural models belonging to ubiquitin receptors Rpn10 and Rpn11. (**C**) Three-dimensional arrangement of the deubiquitinating enzyme Rpn11. (**D**–**F**) Structural prediction of the ATPase subunits Rpt2, Rpt3 and Rpt5 involved in opening the α-gate.

Three-dimensional modeling and structural alignments were conducted to obtain an approximation of the three-dimensional organization of the 19S proteasome subunits of *N. fowleri* and to evaluate their structural homology in comparison with *H. sapiens* (Figure 2).

For the non-ATPase subunits, the ubiquitin receptors Rpn10 and Rpn13 showed approximately 17% identity and 40% similarity (Figure 2A,B). The deubiquitinating enzyme Rpn11 exhibited higher conservation, with 34% identity and 66% similarity (Figure 2C). The ATPase subunits demonstrated greater structural conservation relative to their non-ATPase counterparts. The Rpt2, Rpt3, and Rpt5 subunits presented approximately 38–45% identity and 60–73% similarity when comparing the amoeba sequences with those of *H. sapiens* (Figure 2D–F). These results indicate that, at the structural level, the ATPase components of the 19S proteasome in *N. fowleri* exhibit a higher degree of similarity to the human sequences than the non-ATPase subunits.

### 3.3. N. fowleri Trophozoites Have the Proteins from the ATPase and Non-ATPase Subunits of the 19S Proteasome

**Figure 3:**
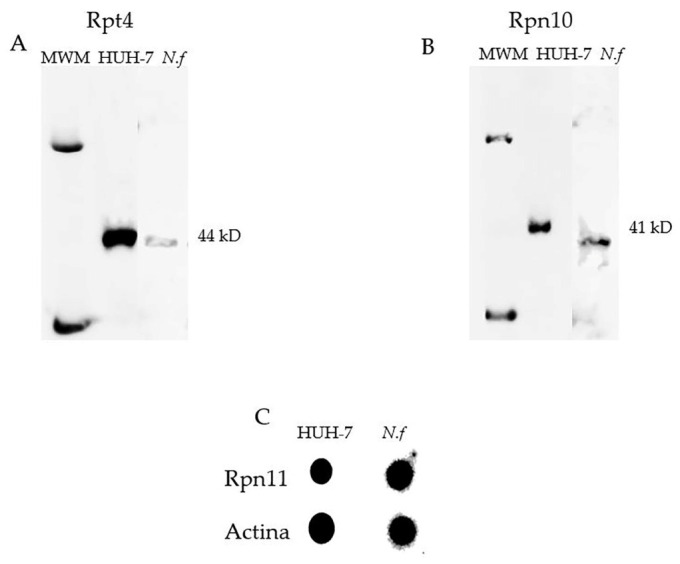
Western blot and dot blot of total extracts of *N. fowleri* and HUH-7 cells. (**A**) Bands that correspond to the Rpt4 ATPase ring subunit of the 19S regulatory particle. (**B**) Bands corresponding to Rpn10, the receptor subunit. (**C**) Dot blot assay of the deubiquitinating enzyme Rpn11 subunit in *N. fowleri* and HUH-7 cells. Actin was used as a loading control.

To determine the presence of 19S proteasome subunits, Western blot assays were performed using total cell extracts from *N. fowleri* trophozoites, with HUH-7 cells used as a positive control. The results demonstrated the presence of the constitutive proteasome subunits Rpt4 and Rpn10 in both amoebae and hepatocarcinoma cells (Figure 3). Immunoblot analysis revealed the presence of the Rpt4 subunit in *N. fowleri*, exhibiting a molecular weight of approximately 44 kDa. A band of the same molecular weight was also detected in HUH-7 cells; however, in these cells, the signal was more pronounced, showing a stronger and broader band compared with that observed in the amoebae (Figure 3A). In addition, the receptor subunit Rpn10 was detected in both organisms, with an apparent molecular weight of approximately 39 kDa in *N. fowleri* and 41 kDa in HUH-7 cells (Figure 3B). We suggest that this difference may be attributed to the smaller protein sequence predicted for the amoebic homolog. In contrast, the deubiquitinating enzyme subunit Rpn11 could not be detected by Western blot analysis in total cell extracts of *N. fowleri*. Therefore, a dot blot assay was performed to evaluate the presence of this protein. Positive signals were obtained for both *N. fowleri* and HUH-7 cells, indicating the presence of the Rpn11 subunit in both samples (Figure 3C).

### 3.4. Intracellular Location of the 19S Proteasome Subunits in N. fowleri

**Figure 4:**
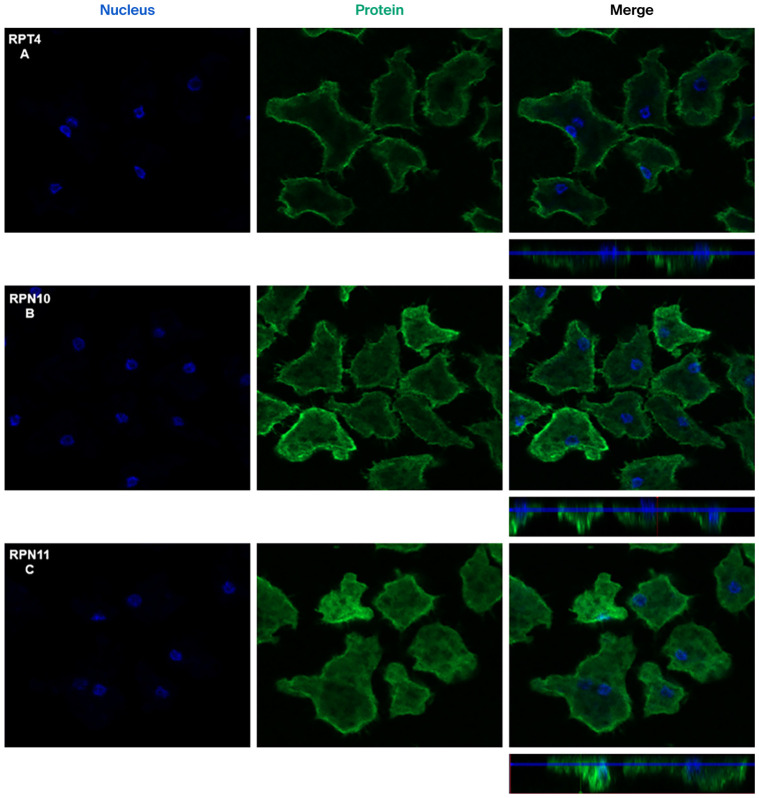
Subcellular location of the 19S proteasome subunits in *N. fowleri* trophozoites. Confocal microscopy images show a predominantly cytosolic localization; however, these proteins are also present in the membrane. (**A**) Rpt4 ATPase subunit. (**B**) Rpn10 proteasome receptor. (**C**) Rpn11 deubiquitinating enzyme. The first images of each protein represent nuclei stained with DAPI (blue), while the different subunits are shown in green. The XZ stack demonstrates that the proteasome subunits surround the nucleus. Images were acquired using a 40× objective lens with an additional 3× zoom.

**Figure 5:**
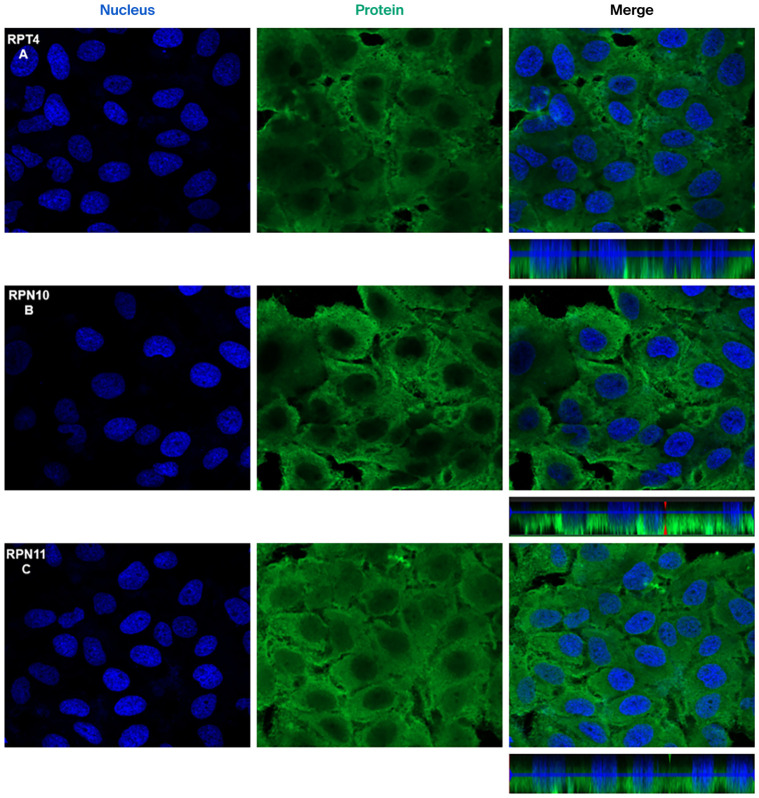
Subcellular location of the 19S proteasome subunits in HUH-7 cells. Confocal microscopy images show a localization primarily in the cytosol and nuclei. (**A**) Rpt4 ATPase subunit. (**B**) Rpn10 proteasome receptor. (**C**) Rpn11 deubiquitinating enzyme. The first images of each protein represent nuclei stained with DAPI (blue), while the different subunits are shown in green. Images were acquired using a 40× objective lens.

To determine the subcellular localization of the 19S proteasome subunits in *N. fowleri* trophozoites, immunofluorescence assays were performed. The results showed that the signals corresponding to the proteins analyzed (Rpt4, Rpn10, and Rpn11) were predominantly distributed in the cytoplasm. Additionally, a spot-like pattern was observed at the membrane and surrounding the nucleus (Figure 4). In contrast, in HUH-7 cells, the signal was detected both in the cytoplasm and inside the nucleus (Figure 5). Because the presence of these proteins within the nucleus of amoebae was not clearly distinguishable, ZX sections from confocal microscopy images were analyzed. These sections revealed that, in *N. fowleri*, the subunits appear localized around the nucleus rather than inside it (Figure 4A–C), whereas in HUH-7 cells, the labeled proteins were observed in the cytoplasm and clearly within the nucleus (Figure 5A–C).

**Figure 6:**
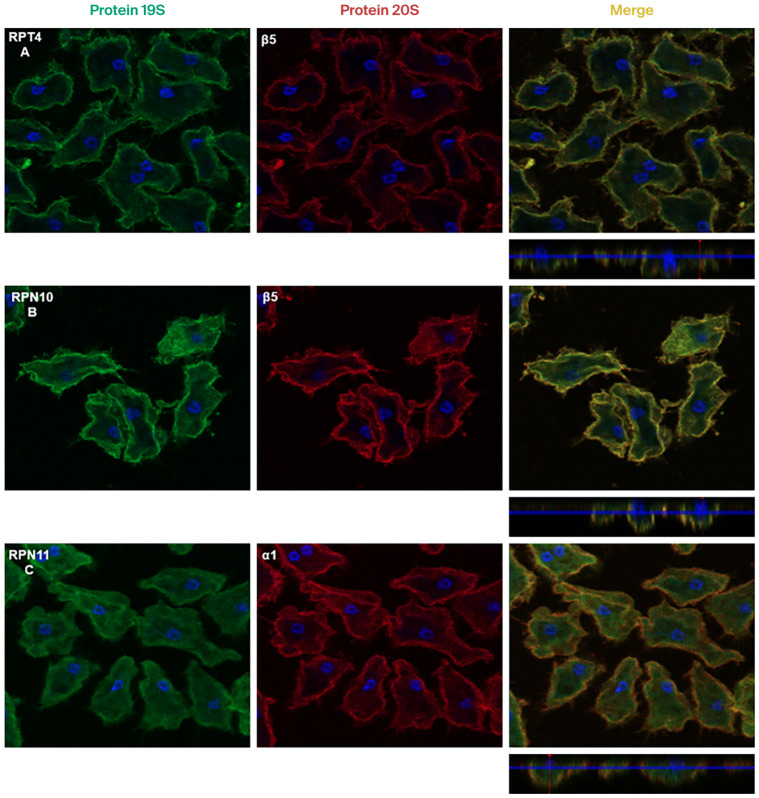
Colocalization of the 19S proteasome with the 20S proteasome in *N. fowleri* trophozoites. Images show primarily cytosolic localization for both fluorochromes; however, these proteins are also present in discrete regions of the membrane. (**A**) Rpt4 ATPase subunit and α1. (**B**) Rpn10 proteasome receptor and α1. (**C**) Rpn11 deubiquitinating enzyme and α1. The nuclei were stained with DAPI (blue); the 19S proteasome subunits are shown in green, the 20S subunit is shown in red, and the merged signal is represented in yellow. The XZ stack demonstrates that the proteasome subunits surround the nucleus. Images were acquired using a 40× lens with an additional 3× zoom.

**Figure 7:**
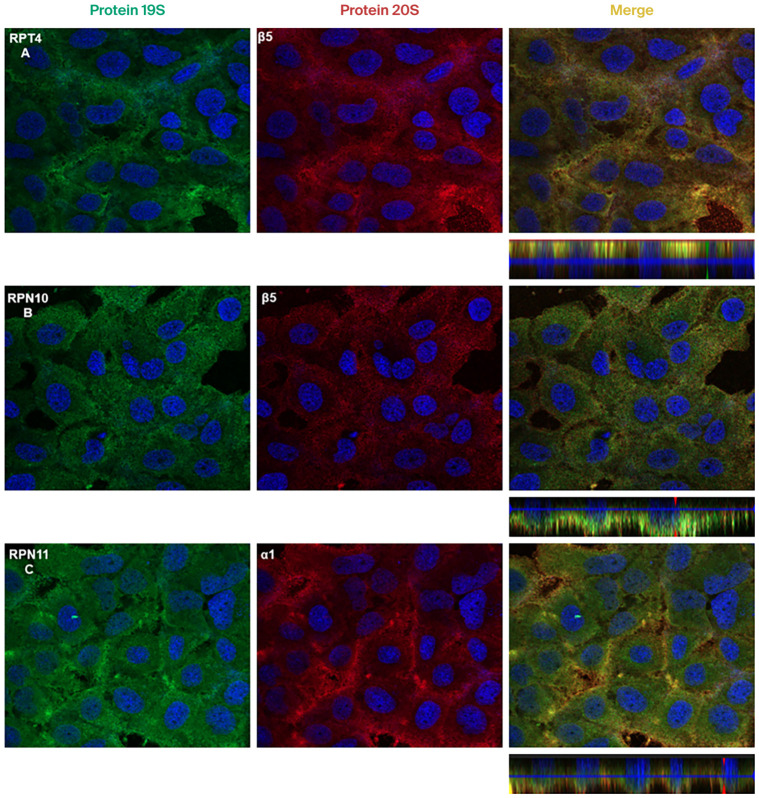
Colocalization of the 19S proteasome with the 20S proteasome in HUH-7 cells. Images show a localization primarily in the cytosol and partially in the nucleus for both fluorochromes. (**A**) Rpt4 ATPase subunit and α1. (**B**) Rpn10 proteasome receptor and α1. (**C**) Rpn11 deubiquitinating enzyme and α1 The nuclei were stained with DAPI (blue); the 19S proteasome subunits are shown in green, the 20S subunit is shown in red, and the merged signal is represented in yellow. The XZ stack demonstrates that the proteasome subunits surround the nucleus. Images were acquired using a 40× lens.

To confirm the association of the 19S and 20S complexes forming the 26S proteasome, double immunolabeling assays were performed. In both organisms, colocalization of the 19S and 20S proteasomes was evident in the merged images of the two fluorochromes. In *N. fowleri*, the signal was mainly localized in the cytoplasm, with spot-like patterns in the membrane and surrounding the nucleus (Figure 6), while in HUH-7 cells, the signal appeared in the cytoplasm and within the nucleus (Figure 7). In both organisms, individual spots corresponding to each fluorochrome—green for 19S and red for 20S—were also detected in the cytoplasm, indicating the presence of unassembled proteasome subunits.

### 3.5. N fowleri Presents Various Proteins Corresponding to Both 26S Proteasome Complexes

To obtain the largest number of proteins related to the 26S proteasome present in the amoeba, the final purified sample was analyzed by mass spectrometry. From the analysis performed on the *N. fowleri* sample, a total of 635 proteins were identified, of which 18 belong to the 26S proteasome (Table 2). However, among the total proteins found, 106 remain uncharacterized in the UniProt database (https://www.uniprot.org/ accessed on 20 January 2026). BLAST-P analysis was conducted on the uncharacterized proteins to determine whether additional proteasome-related proteins were present, revealing one more protein associated with the proteasome. In the case of HUH-7 cells, the analysis yielded 850 proteins, of which 34 belong to this proteolytic complex (Supplementary Table S1).

### 3.6. Electron Microscopic Analysis

**Figure 8:**
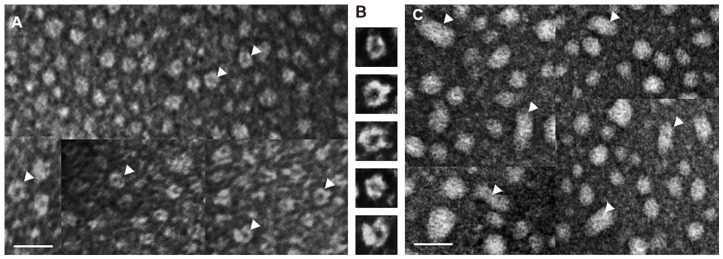
Low-magnification micrograph of the 26S complex in *N. fowleri* negatively stained with uranyl acetate. (**A**) White arrowheads indicate ring-shaped structures with a diameter of approximately 15 nm, characteristic of the 20S catalytic core; scale bar: 1 cm = 30 nm. (**B**) Magnified view of the 20S proteasome to better visualize the ring-like structure and its central pore. (**C**) White arrowheads indicate cylindrical structures approximately 45–50 nm in size, corresponding to the 26S proteasome complex; scale bar: 1 cm = 50 nm.

Electron microscopic analysis was performed to evaluate the 26S proteasome in *N. fowleri.* Figure 8 shows a low-magnification micrograph of the complex negatively stained with uranyl acetate. The enzyme molecule 20S appears as ring-shaped particles with a diameter of approximately 13–15 nm (Figure 8A), corresponding to the familiar view of the 20S proteasome. Various complexes were magnified to better observe the ring-shaped structure of the complex as well as the central pore (Figure 8B). Similarly, some structures resembling the 26S proteasome were also observed, with an average total length of 50 nm and a diameter of approximately 15 nm (Figure 8C).

## 4. Discussion

The 26S proteasome is recognized as the main protease responsible for ubiquitin-dependent intracellular degradation and recycling of short-lived proteins, playing a crucial role in essential cellular functions such as cell cycle regulation, DNA replication, transcription, signal transduction, and stress responses [12,13]. This multicatalytic complex is highly conserved across the three domains of life (Bacteria, Archaea, and Eukarya). In archaea, the PAN–20S (proteasome-activating nucleotidase) complex is present, consisting of 28 subunits forming the 20S core particle together with six regulatory particle (RP) subunits. This assembly is considered an evolutionary precursor of the eukaryotic proteasome. In contrast, bacterial lineages possess the actinobacterial AAA ATPase, which assembles into a ring-shaped complex known as ARC [13,25]. Based on the results obtained in this study, the putative sequences corresponding to the 19S proteasome of N. fowleri were identified, along with their percentages of identity and similarity at both the primary sequence level and in their three-dimensional models. Among these, the ATPase subunits and the deubiquitinating enzyme Rpn11 exhibited the highest degree of homology and structural conservation when compared with the human 19S proteasome. Notably, previous work has demonstrated that within the regulatory particle, the ATPase subunits, together with the non-ATPase Rpn11, are the most conserved components. This has been supported by studies employing annotation-based analyses and similarity searches, in which orthologs of most PA700 (regulatory particle) base and lid subunits were detected across multiple eukaryotic lineages, including sequences highly similar to the six AAA ATPase subunits. Additionally, searches performed using TBLASTN, BLAST-P, and PHI-BLAST revealed that ATPases and Rpn11 consistently yielded very low e-values, suggesting they correspond to true orthologs. These findings support the notion that archaeal PANs represent genuine orthologs of the eukaryotic PSMC (Proteasome 26S Subunit, AAA ATPase) proteins [26]. In higher organisms, it is well established that most subunits of the yeast regulatory particle have homologs in mammals. The Rpt subunits share approximately 66–76% sequence identity between both organisms, whereas the Rpn subunits exhibit identities ranging from 33 to 47%. Notably, Rpn11 displays about 65% identity with its human counterpart [27]. These observations are consistent with the results obtained in this study for N. fowleri. Additionally, two of the non-ATPase subunits analyzed in silico are recognized polyubiquitin receptors. In S. cerevisiae, five ubiquitin receptors involved in proteasome-mediated protein degradation have been described, two of which—Rpn10 and Rpn13—are regulatory subunits of the 26S proteasome and possess orthologs in higher eukaryotes [28]. The proteasome plays a central role in parasitic protozoa, participating in multiple cellular pathways and contributing significantly to the pathogenicity of these organisms. Several parasites undergo morphological transitions that are essential for their infectious cycles, suggesting that the proteasome may be deeply involved in regulating these processes [29]. Although most proteasome studies have traditionally focused on the catalytic core, analyses of the 19S regulatory particle have also been conducted in some protozoa. For instance, in silico studies in species of the Entamoeba genus demonstrated that *E. histolytica*, *E. dispar*, and *E. invadens* encode all components of the ubiquitin–proteasome system (UPS). This conclusion was supported by similarity analyses of sequences and domains using BLAST against genome sequences retrieved from the NCBI database, revealing comparable levels of complexity across these species [30]. Within the genus Trypanosoma, studies in *T. brucei* showed that all six genes homologous to the Rpt1–Rpt6 ATPase subunits are present, as demonstrated through the cloning of six corresponding cDNAs. To evaluate the presence of the 26S proteasome and determine whether it remains intact or dissociates into the 20S core particle and the 19S regulatory particle, cell lysates were fractionated using a Superose 6 column. The resulting fractions were analyzed by Western blot with rabbit antisera specific for Rpt2, Rpt5, Rpt6, and 20S subunits, confirming the presence of these components [31].

Similarly, the presence of the 26S proteasome has also been documented in *T. cruzi*. In this parasite, a high-molecular-weight complex of approximately 1400 kDa, consistent with the expected size and composition of the 26S proteasome, was identified. Chromatographic purification of the complex from epimastigotes, followed by SDS-PAGE and Western blot analyses, revealed roughly 30 proteins ranging from 25 to 110 kDa. Members of the 26S AAA ATPase family were detected using specific antibodies, further supporting the identification of the proteasomal complex [32]. In the case of the 19S proteasome in *N. fowleri*, the dot blot assay yielded positive signals indicating the presence of the analyzed subunits. It is important to note that the presence of the 20S proteasome in this amoeba had previously been demonstrated by our group [33]. Through bioinformatic analyses, the 20S catalytic core was identified in both *N. fowleri* and *N. gruberi*. Moreover, using Western blot, dot blot, and enzymatic activity assays with specific substrates, the structural and catalytic components of the complex were detected, and confocal microscopy revealed a predominantly cytoplasmic localization [33].

Consistent with those findings, our confocal microscopy results show that the 19S proteasome is primarily localized in the cytosol, with additional signal detected in regions adjacent to the nucleus in amoeba trophozoites. In mammalian cells, the 26S proteasome is known to reside in the nucleus, cytoplasm, and endoplasmic reticulum; however, its distribution can vary depending on the cell type and stage of development [34,35]. In some protozoa, the 26S proteasome appears to exhibit variations in subcellular distribution. In *Toxoplasma* and *E. histolytica*, the complex is primarily restricted to the cytosol, whereas in *T. cruzi*, it has been reported in the nucleus, cytoplasm, and kinetoplasts [10,36]. Consistent with the localization observed in this study, both the 19S and the previously described 20S complexes in *N. fowleri* are predominantly found in the cytoplasm and in regions surrounding the nucleus. Using antibodies against the 20S subunits, we also assessed the spatial correlation of the 19S and 20S complexes to infer the formation of the 26S proteasome. Nevertheless, individual foci corresponding to each complex were still observed in both amoeba trophozoites and HUH-7 cells. This phenomenon may occur because certain subunits can function as part of proteasome subcomplexes or even independently [37]. The findings of this study indicate that these proteasome subunits are present in some regions of the plasma membrane. To date, this complex has not been reported in this region of pathogenic protozoa; however, proteasomes have been associated with several cellular locations beyond the cytosol and nucleus, including the endoplasmic reticulum, nucleolus, intermediate filaments, and both the nuclear and plasma membranes. In these contexts, the proteasome contributes to processes such as antigen processing, the degradation of unassembled ribosomal proteins, and the turnover of cytoskeletal network components linked to the nuclear and plasma membranes. These roles suggest that the proteasome may participate in essential cellular functions related to cell proliferation, cell cycle progression, differentiation, and morphogenesis [34]. To further substantiate the presence of the 26S proteasome in amoebae, a purification strategy followed by quantitative mass spectrometry was employed. This approach enabled the identification of 18 proteins associated with the 26S proteasome, including seven subunits corresponding to the 19S regulatory particle, ten to the 20S core particle, and one chaperone involved in complex assembly. These results support the notion that amoebae contain the essential components required for 26S proteasome function. In contrast, analysis of HUH-7 cells yielded a broader set of 34 proteasome-related proteins, comprising 17 subunits of the 19S particle, 15 of the 20S particle, and two activator proteins, reflecting a more extensive proteasomal repertoire typical of human cells. Comparable findings were reported in 2007, when the human 26S proteasome was characterized through liquid chromatography–tandem mass spectrometry, leading to the identification of 14 subunits of the 20S core and 21 subunits of the 19S regulatory particle, providing further insight into the structural organization and degradation pathway of this complex [38]. The purification strategy applied in this study mirrors the approach previously used to confirm that the human 26S proteasome is composed of numerous proteins and associated regulatory components. This method requires the inclusion of high concentrations of ATP and glycerol to maintain complex stability, followed by ultracentrifugation. Nevertheless, rapid handling remains essential, as the 26S proteasome is known to be highly unstable [39]. Despite the methodological constraints, electron microscopy allowed the visualization of representative 20S and 26S proteasome particles in *N. fowleri*. The observed structures exhibited a circular configuration with a small central pore measuring approximately 13–15 nm in diameter. This morphology resembles that described in rat liver, where a ring-shaped enzymatic complex of roughly 160 Å in diameter with a central opening of 10–20 Å was documented [40]. The structural features observed for the 26S proteasome are consistent with previously reported descriptions of this complex. In our analysis, the 26S particle appeared as a cylindrical structure measuring roughly 50 nm in total length, with a central region of about 15 nm in diameter corresponding to the 20S subcomplex. This morphology closely parallels that described in rat liver preparations, where dumbbell-like particles of approximately 45–50 nm (n = 50) were documented. Those structures consisted of a central rectangular segment (around 13 × 17 nm) flanked by two larger, irregular rectangles of approximately 20 × 13 nm. The predominance of these dumbbell-shaped particles, along with the relatively well-preserved symmetry observed under the electron microscope, supports the interpretation that they represent intact or near-intact 26S proteasomes. Based on these features, previous authors proposed that such structures most accurately reflect the architecture of the 26S complex [41]. The micrograph obtained here appears somewhat diffuse, and slight deformation of the 20S complex can be detected. Such alterations are common during electron microscopy sample preparation, as exposure of the inner cavity to solvent can facilitate dye penetration, yielding an image suggestive of an annular or distorted structure. In the same way, the appearance of the 26S complex as a cylindrical dumbbell—with a long, narrow central cylinder and two shorter but wider cylinders at each end—is noteworthy. This arrangement results in the terminal regions having a greater diameter than the central portion. It is important to consider, however, that the drying process required for electron microscopy likely caused a flattening of the terminal domains, potentially altering the precise geometry at the interfaces between the three structural regions. Similar observations and methodological considerations have been reported previously [40,41]. It is essential to investigate the specific functions of the 19S proteasome in these organisms, its potential role in virulence, the substrate proteins recognized and degraded by the 26S proteasome, and whether it could serve as a potential therapeutic target against PAM.

## 5. Conclusions

**Figure 9:**
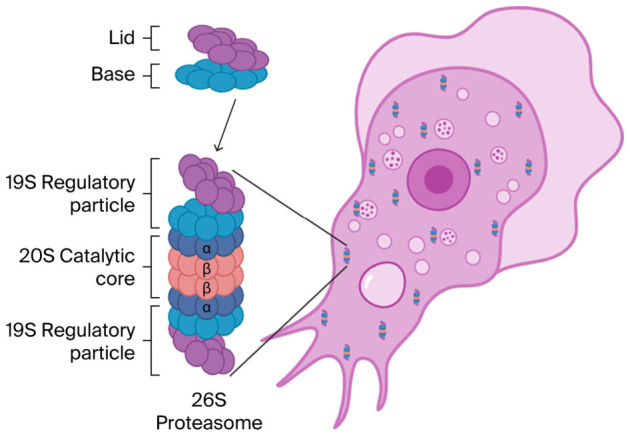
Representative image showing the presence of the 26S proteasome in *N. fowleri*. This complex is composed of two subcomplexes: the 20S catalytic core and the 19S regulatory particle, which is further divided into the base and lid. Based on the results obtained in this study for the 19S proteasome, together with previously published data for the 20S proteasome, both complexes are present in the trophozoites of *N. fowleri*. The 26S proteasome is primarily localized in the cytoplasm; however, it is also observed surrounding the nucleus and may be present in regions of the plasma membrane.

In this study, we demonstrated that *N. fowleri* possesses the 19S proteasome, composed of both ATPase and non-ATPase subunits, with a primarily cytoplasmic localization. Along with the previously described 20S proteasome, these findings support the assembly of the 26S proteasome in this amoeba (Figure 9, created with https://BioRender.com), as confirmed by mass spectrometry, electron microscopy, and confocal microscopy.

## Figures and Tables

**Table 1:** Percentages of identities and similarities among proteasome subunit sequences of *N. fowleri* compared to *H. sapiens*.

*H. sapiens*–*N. fowleri*			
Sequence	% Identity (NCBI)	% Similarity (NCBI)	Value of e
Rpn10	40	58	7 × 10^−67^
Rpn13	55	71	3 × 10^−35^
Rpn11	75	85	7 × 10^−164^
Rpt2	74	87	0.0
Rpt3	79	91	0.0
Rpt5	70	85	0.0

The multiple sequence alignment performed with NCBI BLAST-P revealed the percentage of identity between the *N. fowleri* sequence and the template sequence (*H. sapiens*), as well as the number of amino acids classified as similar between both sequences.

**Table 2:** Proteins related to the 26S proteasome found in *N. fowleri* through mass spectrometry analysis.

No.	Protein Accession	Protein Description	Mass (Da)
1	A0A6A5B0P3	26S proteasome regulatory subunit RPN1 OS = Naegleria fowleri OX = 5763 GN = FDP41_008657 PE = 3 SV = 1	107,351
2	A0A6A5BYT3	AAA+ ATPase domain-containing protein OS = Naegleria fowleri OX = 5763 GN = FDP41_001928 PE = 3 SV = 1	35,385
3	A0A6A5BRX1	AAA+ ATPase domain-containing protein OS = Naegleria fowleri OX = 5763 GN = FDP41_003228 PE = 3 SV = 1	50,731
4	A0A6A5BBV0	AAA+ ATPase domain-containing protein OS = Naegleria fowleri OX = 5763 GN = FDP41_005397 PE = 3 SV = 1	46,026
5	A0A6A5CG27	AAA+ ATPase domain-containing protein OS = Naegleria fowleri OX = 5763 GN = FDP41_007475 PE = 3 SV = 1	45,323
6	A0A6A5B4X9	AAA+ ATPase domain-containing protein OS = Naegleria fowleri OX = 5763 GN = FDP41_008641 PE = 3 SV = 1	48,555
7	A0A6A5C565	AAA+ ATPase domain-containing protein OS = Naegleria fowleri OX = 5763 GN = FDP41_012261 PE = 3 SV = 1	50,811
8	A0A6A5C1E9	Proteasome alpha-type subunits domain-containing protein OS = Naegleria fowleri OX = 5763 GN = FDP41_001386 PE = 3 SV = 1	27,377
9	A0A6A5AZ25	Proteasome alpha-type subunits domain-containing protein OS = Naegleria fowleri OX = 5763 GN = FDP41_009990 PE = 3 SV = 1	25,637
10	A0A6A5C2Z4	Proteasome assembly chaperone 1 OS = Naegleria fowleri OX = 5763 GN = FDP41_012910 PE = 4 SV = 1	40,510
11	A0A6A5BQ66	Proteasome subunit alpha-type OS = Naegleria fowleri OX = 5763 GN = FDP41_005852 PE = 3 SV = 1	27,699
12	A0A6A5BIR7	Proteasome subunit alpha-type OS = Naegleria fowleri OX = 5763 GN = FDP41_006226 PE = 3 SV = 1	28,371
13	A0A6A5BHW7	Proteasome subunit alpha-type OS = Naegleria fowleri OX = 5763 GN = FDP41_008858 PE = 3 SV = 1	29,007
14	A0A6A5BV33	Proteasome subunit alpha-type OS = Naegleria fowleri OX = 5763 GN = FDP41_011804 PE = 3 SV = 1	30,076
15	A0A6A5BPD8	Proteasome subunit beta OS = Naegleria fowleri OX = 5763 GN = FDP41_001036 PE = 3 SV = 1	27,503
16	A0A6A5BPM0	Proteasome subunit beta OS = Naegleria fowleri OX = 5763 GN = FDP41_004823 PE = 3 SV = 1	32,972
17	A0A6A5BSB3	Proteasome subunit beta OS = Naegleria fowleri OX = 5763 GN = FDP41_005126 PE = 3 SV = 1	28,645
18	A0A6A5CEI7	Proteasome subunit beta OS = Naegleria fowleri OX = 5763 GN = FDP41_007915 PE = 3 SV = 1	23,524

Mass spectrometry analysis identified 18 proteins associated with the 26S proteasome. Among these, 7 correspond to subunits of the 19S regulatory complex, 10 are components of the 20S catalytic core, and one protein is classified as a proteasome chaperone. The table summarizes the number of UniProt accessions available for each protein, the name under which it is registered in the database, and its corresponding molecular weight.

## Data Availability

The original contributions presented in this study are included in the article/Supplementary Material. Further inquiries can be directed to the corresponding author.

## Supplementary Materials

The following supporting information can be downloaded at https://www.mdpi.com/article/10.3390/microorganisms14061277/s1, Figure S1: Purification of the proteasome.; Table S1: Proteins related to 26S proteasome found in HUH-7 through mass spectrometry analysis.

## Abbreviations

The following abbreviations are used in this manuscript:

FLA	Free-living amoebae
CNS	Central nervous system
PAM	Primary amebic meningoencephalitis
AmB	Amphotericin B
HUH-7	Human hepatocyte-derived carcinoma cell line
RP	Regulatory particle
MS	Mass spectrometry
PAN–20S	proteasome-activating nucleotidase
UPS	Ubiquitin–proteasome system
