# Supplementary material for: Identification and Characterization of the Regulatory Particle of Proteasome 19S and Its Correlation with Proteasome 26S in Trophozoites of Naegleria fowleri
Source: Microorganisms. 2026 Jun 5;14(6):1277. doi: 10.3390/microorganisms14061277 (PMC13302919; doi:10.3390/microorganisms14061277)
Supplement: Supplementary file 1 [file microorganisms-14-01277-s001.zip › microorganisms-4332263-supplementary.pdf]

Supplementary Table S1. Proteins related to 26S proteasome found in HUH-7 through mass spectrometry analysis

No.	Protein Accession	Protein Description	Mass (Da)
1	A0A7I2V2K8	26S proteasome non-ATPase regulatory subunit 1 OS=Homo sapiens OX=9606 GN=PSMD1 PE=1 SV=1	98,960
2	O00231	26S proteasome non-ATPase regulatory subunit 11 OS=Homo sapiens OX=9606 GN=PSMD11 PE=1 SV=3	47,464
3	O00232	26S proteasome non-ATPase regulatory subunit 12 OS=Homo sapiens OX=9606 GN=PSMD12 PE=1 SV=3	52,904
4	Q9UNM6	26S proteasome non-ATPase regulatory subunit 13 OS=Homo sapiens OX=9606 GN=PSMD13 PE=1 SV=2	42,945
5	O00487	26S proteasome non-ATPase regulatory subunit 14 OS=Homo sapiens OX=9606 GN=PSMD14 PE=1 SV=1	34,577
6	Q13200	26S proteasome non-ATPase regulatory subunit 2 OS=Homo sapiens OX=9606 GN=PSMD2 PE=1 SV=3	100,200
7	O43242	26S proteasome non-ATPase regulatory subunit 3 OS=Homo sapiens OX=9606 GN=PSMD3 PE=1 SV=2	60,978
8	Q15008	26S proteasome non-ATPase regulatory subunit 6 OS=Homo sapiens OX=9606 GN=PSMD6 PE=1 SV=1	45,531
9	P51665	26S proteasome non-ATPase regulatory subunit 7 OS=Homo sapiens OX=9606 GN=PSMD7 PE=1 SV=2	37,025
10	P62333	26S proteasome regulatory subunit 10B OS=Homo sapiens OX=9606 GN=PSMC6 PE=1 SV=1	44,173
11	P62191	26S proteasome regulatory subunit 4 OS=Homo sapiens OX=9606 GN=PSMC1 PE=1 SV=1	49,185
12	P43686	26S proteasome regulatory subunit 6B OS=Homo sapiens OX=9606 GN=PSMC4 PE=1 SV=2	47,366
13	C9JX88	26S proteasome regulatory subunit 7 OS=Homo sapiens OX=9606 GN=PSMC2 PE=1 SV=1	48,263
14	A0A994J6V8	26S proteasome regulatory subunit 8 OS=Homo sapiens OX=9606 GN=PSMC5 PE=1 SV=1	42,649
15	Q99460-2	Isoform 2 of 26S proteasome non-ATPase regulatory subunit 1 OS=Homo sapiens OX=9606 GN=PSMD1	105,836
16	P25788-2	Isoform 2 of Proteasome subunit alpha type-3 OS=Homo sapiens OX=9606 GN=PSMA3	28,433
17	H3BT36	Proteasome 20S subunit alpha 2 OS=Homo sapiens OX=9606 GN=PSMA2 PE=1 SV=1	5,011
18	R4GNH3	Proteasome 26S subunit_ ATPase 3 OS=Homo sapiens OX=9606 GN=PSMC3 PE=1 SV=1	47,352

19	J3KRP2	Proteasome 26S subunit_ ATPase 5 (Fragment) OS=Homo sapiens OX=9606 GN=PSMC5 PE=1 SV=1	27,195
20	Q14997	Proteasome activator complex subunit 4 OS=Homo sapiens OX=9606 GN=PSME4 PE=1 SV=2	211,334
21	B3KQ25	Proteasome activator subunit 3 OS=Homo sapiens OX=9606 GN=PSME3 PE=1 SV=1	22,455
22	H0YMZ1	Proteasome subunit alpha type (Fragment) OS=Homo sapiens OX=9606 GN=PSMA4 PE=1 SV=8	24,526
23	G3V5Z7	Proteasome subunit alpha type OS=Homo sapiens OX=9606 GN=PSMA6 PE=1 SV=1	28,147
24	P25786	Proteasome subunit alpha type-1 OS=Homo sapiens OX=9606 GN=PSMA1 PE=1 SV=1	29,556
25	P25787	Proteasome subunit alpha type-2 OS=Homo sapiens OX=9606 GN=PSMA2 PE=1 SV=2	25,899
26	P28066	Proteasome subunit alpha type-5 OS=Homo sapiens OX=9606 GN=PSMA5 PE=1 SV=3	26,411
27	O14818	Proteasome subunit alpha type-7 OS=Homo sapiens OX=9606 GN=PSMA7 PE=1 SV=1	27,887
28	P20618	Proteasome subunit beta type-1 OS=Homo sapiens OX=9606 GN=PSMB1 PE=1 SV=2	26,489
29	P49721	Proteasome subunit beta type-2 OS=Homo sapiens OX=9606 GN=PSMB2 PE=1 SV=1	22,836
30	P49720	Proteasome subunit beta type-3 OS=Homo sapiens OX=9606 GN=PSMB3 PE=1 SV=2	22,949
31	P28070	Proteasome subunit beta type-4 OS=Homo sapiens OX=9606 GN=PSMB4 PE=1 SV=4	29,204
32	P28074	Proteasome subunit beta type-5 OS=Homo sapiens OX=9606 GN=PSMB5 PE=1 SV=3	28,480
33	P28072	Proteasome subunit beta type-6 OS=Homo sapiens OX=9606 GN=PSMB6 PE=1 SV=4	25,358
34	Q99436	Proteasome subunit beta type-7 OS=Homo sapiens OX=9606 GN=PSMB7 PE=1 SV=1	29,965

Through mass spectrometry analysis, a broader set of 34 proteasome-related proteins was identified in HUH-7 cells. This set comprised 17 subunits of the 19S particle, 15 subunits of the 20S particle, and two activator proteins, thus providing an expanded repertoire of the 26S proteasome. The table summarizes the number of UniProt accessions available for each protein, the name under which it is registered in the database, and its corresponding molecular weight.

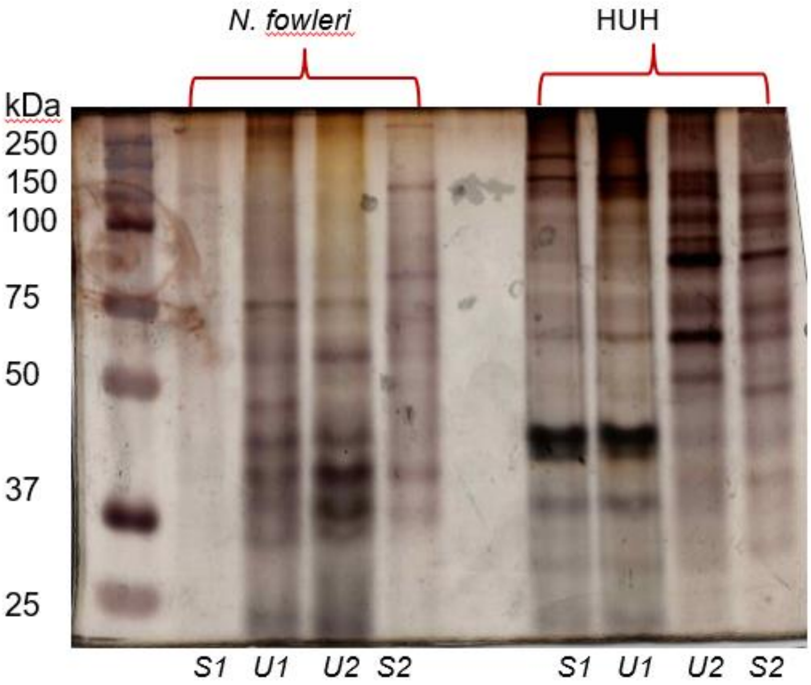

Figure S1. Purification of the proteasome. The gel shows the protein pools of *N. fowleri* and HUH cells obtained at each step of the purification procedure, where S1 corresponds to the sonication pellet, U1 to ultracentrifugation pellet 1, U2 to ultracentrifugation pellet 2, and S2 to the ultracentrifugation supernatant. The image corresponds to a 12% SDS–PAGE gel loaded with 15 μg of protein and stained with silver.
